# Neuroprotective Potential of SGLT2 Inhibitors in Animal Models of Alzheimer’s Disease and Type 2 Diabetes Mellitus: A Systematic Review

**DOI:** 10.3390/ph19010166

**Published:** 2026-01-16

**Authors:** Azim Haikal Md Roslan, Tengku Marsya Hadaina Tengku Muhazan Shah, Shamin Mohd Saffian, Lisha Jenny John, Muhammad Danial Che Ramli, Che Mohd Nasril Che Mohd Nassir, Mohd Kaisan Mahadi, Zaw Myo Hein

**Affiliations:** 1 Centre for Drug and Herbal Development, Faculty of Pharmacy, Universiti Kebangsaan Malaysia, Jalan Raja Muda Abdul Aziz, Kuala Lumpur 50300, Malaysia; p154166@siswa.ukm.edu.my (A.H.M.R.); tengkumarsya76@gmail.com (T.M.H.T.M.S.); 2 Centre for Quality Management of Medicine, Faculty of Pharmacy, Universiti Kebangsaan Malaysia, Jalan Raja Muda Abdul Aziz, Kuala Lumpur 50300, Malaysia; shamin@ukm.edu.my; 3 Department of Basic Medical Sciences, College of Medicine, Ajman University, Ajman P.O. Box 346, United Arab Emirates; l.john@ajman.ac.ae; 4 Department of Diagnostic and Allied Health Science, Faculty of Health and Life Sciences, Management and Science University, Shah Alam 40150, Malaysia; muhddanial_cheramli@msu.edu.my; 5 Department of Anatomy and Physiology, School of Basic Medical Sciences, Faculty of Medicine, Universiti Sultan Zainal Abidin, Kuala Terengganu 20400, Malaysia; nasrilnassir@unisza.edu.my

**Keywords:** Alzheimer’s disease, type 2 diabetes mellitus, spatial memory function, Morris Water Maze test, sodium–glucose cotransporter-2 inhibitors

## Abstract

**Background:** Alzheimer’s disease (AD) features progressive cognitive decline and amyloid-beta (Aβ) accumulation. Insulin resistance in type 2 diabetes mellitus (T2DM) is increasingly recognised as a mechanistic link between metabolic dysfunction and neurodegeneration. Although sodium–glucose cotransporter-2 inhibitors (SGLT2is) have established glycaemic and cardioprotective benefits, their neuroprotective role remains less well defined. **Objectives:** This systematic review examines animal studies on the neuroprotective effects of SGLT2i in T2DM and AD models. **Methods:** A literature search was conducted across the Web of Science, Scopus, and PubMed databases, covering January 2014 to November 2024. Heterogeneity was assessed with I^2^, and data were pooled using fixed-effects models, reported as standardised mean differences with 95% confidence intervals. We focus on spatial memory performance as measured by the Morris Water Maze (MWM) test, including escape latency and time spent in the target quadrant, as the primary endpoints. The secondary endpoints of Aβ accumulation, oxidative stress, and inflammatory markers were also analysed and summarised. **Results:** Twelve studies met the inclusion criteria for this review. A meta-analysis showed that SGLT2i treatment significantly improved spatial memory by reducing the escape latency in both T2DM and AD models. In addition, SGLT2i yielded a significant improvement in spatial memory, as indicated by an increased target quadrant time for both T2DM and AD. Furthermore, SGLT2i reduced Aβ accumulation in the hippocampus and cortex, which met the secondary endpoint; the treatment also lessened oxidative stress and inflammatory markers in animal brains. **Conclusions:** Our findings indicate that SGLT2is confer consistent neuroprotective benefits in experimental T2DM and AD models.

## 1. Introduction

Alzheimer’s disease (AD) is a progressive neurodegenerative disorder characterised by a decline in cognitive functions; it primarily affects individuals older than 65 years. AD can progress from short-term memory loss to severe functional disability that leads to death within several years of diagnosis [1]. The substantial decline in cognitive abilities is generally described as dementia. AD accounts for between 60% and 80% of all dementia cases and greatly diminishes people’s quality of life. The disease also imposes a vast financial burden on healthcare systems [2]. According to global data from the World Health Organisation (WHO), approximately 55 million people worldwide lived with dementia in 2021, and the number is projected to increase to 139 million by 2050 [3]. In Malaysia alone, an estimated 204,000 to 264,000 elderly individuals were affected by AD in 2020, and the WHO expected the number to increase by 312% by 2050. These figures underscore the urgent need for early intervention [4].

The key pathological hallmarks of AD centre on two main features: the formation of beta-amyloid (Aβ) plaques and neurofibrillary tangles (NFTs) in brain regions involved in memory [5]. Elevated inflammatory markers in patients further suggest a prominent role of neuroinflammation in the pathogenesis of AD [6]. Multiple factors, including genetics, ageing, lifestyle, and environmental influences, are thought to contribute to AD development [5]. This multifactorial aetiology has made the development of effective treatment strategies particularly challenging. The U.S. Food and Drug Administration (FDA) has approved several symptomatic therapies for AD, including N-methyl-D-aspartate receptor (NMDAR) antagonists and cholinesterase inhibitors. However, these agents provide only limited, temporary symptomatic relief by slowing cognitive decline, and no curative treatment is currently available [7]. Monoclonal antibodies (MAbs) can effectively reduce Aβ pathology in patients with AD [5]. Nevertheless, MAb therapy is frequently associated with serious adverse events, such as amyloid-related imaging abnormalities, cerebral oedema, and intracranial haemorrhage [8,9].

This failure highlights the need for treatments to address the complex underlying mechanisms of the disease rather than solely managing its symptoms. Another disease that shares substantial pathological mechanisms with AD is type 2 diabetes mellitus (T2DM), a metabolic disorder characterised by insulin resistance [10,11]. T2DM is often associated with low-grade chronic inflammation, which leads to peripheral insulin resistance in tissues [12]. Similarly, AD patients display inflamed brain tissue and impaired neuronal insulin signalling, which exacerbate the neuronal dysfunction [13,14]. Impaired insulin signalling in the brain contributes to the accumulation of extracellular amyloid-beta (Aβ) plaques and tau-based neurofibrillary tangles (NFTs)—which are two hallmark features of AD pathology [15]. About 60% to 70% of individuals with T2DM similarly experience cognitive decline, which indicates the link between metabolic dysregulation and neurodegeneration [10,16].

Repurposing existing drugs that possess established safety profiles may present a promising alternative for AD treatment. Given the shared pathological pathways between AD and T2DM, there is growing interest in exploring T2DM medications as potential therapeutic agents for AD. For example, sodium–glucose cotransporter-2 inhibitors (SGLT2i) are a class of drug that block glucose reabsorption in the kidney, causing it to be excreted in urine, thereby lowering blood sugar level [17]. The SGLT2i drugs include empagliflozin, canagliflozin, dapagliflozin, and ertugliflozin. Beyond their metabolic actions, SGLT2is may influence several key neurodegenerative pathways [18,19,20,21]. Emerging evidence indicates that SGLT2is may also exert neuroprotective effects, which are probably associated with the expression of SGLT2 receptors in the brain. Studies have shown significant SGLT2 receptor expression in hippocampal and cerebellar regions that are critical for memory regulation and in endothelial cells of the blood–brain barrier (BBB) [22,23,24]. SGLT2 receptors are also present in microglia, and SGLT2i may modulate the inflammatory response in these cells [25].

Despite the growing interest in the potential of SGLT2i to mitigate cognitive decline, clinical data on AD patients remain scarce. The only randomised controlled trial designed to evaluate the mental effects of SGLT2i in AD patients is trial NCT03801642, conducted by the University of Kansas Medical Centre. Its full results have not yet been published. As such, the majority of the mechanistic insights have been gained from preclinical data. However, the potential role of SGLT2is in preventing or treating AD has not been systematically or comprehensively assessed. Hence, there is an important gap in the current body of evidence.

In this paper, we present a systematic review and meta-analysis of the literature on the effects of SGLT2i in animal models of T2DM and AD. Our primary focus is on evaluating spatial memory function by analysing escape latency and time spent in the target quadrant during the Morris Water Maze (MWM) test. Additionally, we assess the efficacy of SGLT2i in reducing Aβ accumulation and inflammatory markers in the brains of the animal models.

## 2. Methods

This article follows the guidelines outlined in the Preferred Reporting Items for Systematic Reviews and Meta-Analyses (PRISMA 2020) [26]. The study protocol was registered on the International Prospective Register of Systematic Reviews (PROSPERO) under the identification code CRD420251061359. The record is publicly available at: https://www.crd.york.ac.uk/PROSPERO/view/CRD420251061359 (accessed on 8 January 2026).

### 2.1. Search Strategy

Articles published during the 10-year period from January 2014 until December 2024 were identified through the PubMed, Web of Science, and Scopus electronic databases. We used keywords and terms from the Medical Subject Headings. The keywords were “sodium-glucose cotransporter-2 inhibitor OR empagliflozin OR dapagliflozin OR SGLT2 inhibitor OR SGLT2i” AND “Alzheimer OR cognitive decline OR memory decline OR MWMT OR amyloid beta OR Morris water maze test OR inflammation” AND “diabetes OR insulin resistance OR metabolic syndrome” (Supplementary Materials S1 and S2). All articles from the search results were imported into the reference manager EndNote, and any duplicates were removed.

### 2.2. Inclusion and Exclusion Criteria

Eligible studies satisfied the following inclusion criteria: (1) rodent with clear genetic origin; (2) AD or T2DM model; (3) study included an SGLT2i group and a control group, administered by any route, with the groups being independent; (4) outcomes were either escape latency and time spent in the target quadrant of the MWM test or Aβ accumulation; (5) study was published in English and the full text was accessible.

The following studies were excluded: (1) cell model or non-rodent; (2) studies did not assess the outcomes of interest; (3) we could not access the full text, review, meta-analyses, case report, editorial, abstract, letter, and/or comments; and (4) the paper was not published in English. All records and additional full texts were reviewed after the inclusion and exclusion criteria had been applied.

### 2.3. Data Extraction

The following data were extracted from the included studies: (1) the year of publication and the first author’s name; (2) basic details of the animal models (species, sex, age, weight, and total number); (3) the method of inducing AD or T2DM; (4) intervention characteristics (SGLT2i dose, duration, and administration route); and (5) the outcomes that were evaluated (MWM test, Aβ level and inflammation).

If data were presented only graphically, we used WebPlotDigitizer (version 4.8) to extract numerical values from the graphs. Each figure was calibrated by assigning two reference points on both the x- and y-axes, which is a standard procedure to ensure accurate scaling; this approach has been validated and typically yields extraction errors of less than 2% [27]. A.H.M.R. and T.M.H. independently performed the data extraction using a designated Microsoft Excel file. If disagreement occurred between the first two authors, the third author, M.K.M., and the fourth author, S.M.S., were consulted.

### 2.4. Quality Assessment Method

We evaluated the quality of the included studies based on the Collaborative Approach to Meta-Analysis and Review of Animal Data from Experimental Studies (CAMARADES) research-quality checklist [28]. There were 10 evaluation categories, and the paper was scored 1 point if it met the criteria in a category. Hence, each study could receive a maximum score of 10 points.

### 2.5. Statistical Analysis

A meta-analysis was conducted using R (version 4.1.1) using the meta and metafor packages. Outcomes were treated as continuous data. Given the variability in experimental models and study designs, a random effects model was applied to pool the effect sizes across studies. We employed the standardised mean difference (SMD) to assess the magnitude of treatment effects. We set the confidence interval (CI) to 95%, and *p*-values below 0.05 were regarded as statistically significant. The Q test and I^2^ test were used to evaluate heterogeneity. Furthermore, we assessed the publication bias among the included studies by Egger’s test and visualised the results as a funnel plot. Additionally, trim-and-fill analyses were performed in R to explore the potential impact of small study effects.

## 3. Results

### 3.1. Literature Screening

**Figure 1:**
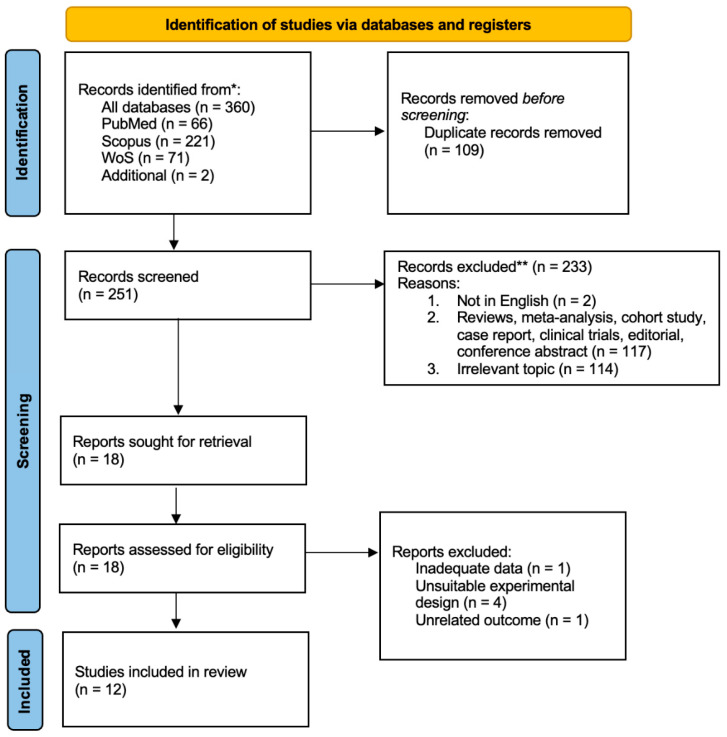
PRISMA flow diagram. * Records excluded after title and abstract screening. ** Records excluded after full-text assessment.

Our search strategy returned 360 papers in total. This includes 66 papers, 221 papers, and 71 papers from PubMed, Scopus, and Web of Science, respectively, and we manually added two studies. We then removed 109 duplicates. Among the remaining pool, we selected 251 articles based on their titles and abstracts. After assessing the relevance of these texts, we excluded 233 of them; our shortlist thus comprised 18 articles for in-depth screening of their full texts. Finally, 12 original articles met all our inclusion criteria and were included in the analyses. Figure 1 illustrates the study selection process.

### 3.2. Characteristics of Reviewed Studies

Table 1 describes the characteristics of the 12 included studies, which were all published between 2014 and 2024. The studies used two types of animals, namely rats (seven studies) and mice (five studies). The group sizes ranged from six to 12 animals per study. All studies described the species of the experimental animals, including two in db/db mice [20,29], three in C57BL/6 mice [30,31,32], six in Wistar rats [21,33,34,35,36,37], one in APP/PS1 mice [20], and one in Swiss rats [38].

AD models were established in four studies through various methods, including intracerebroventricular streptozotocin (icv-STZ) [33], aluminium chloride (AlCl_3_) [38], and ovariectomised/d-galactose [34]. Two studies induced a pharmacological model of AD using scopolamine [35,37]. Another study employed cadmium to induce AD-like symptoms [36]. Among the T2DM studies, three employed streptozotocin induction [21,31,32] and one study fed mice a high-fructose diet to induce the T2DM model [30].

Two studies experimented with two doses of SGLT2i in different groups. The 2 doses are specifically from Borikar, S., N. and Jain [37] and Samman, Selim, El Fayoumi, El-Sayed, Mehanna and Hazem [38] that investigated 2 different doses of SGLT2i. By splitting them into 2 different group is to allow dose-specific effects if clinically relevant. Of the 12 articles we reviewed, five studied empagliflozin [20,29,30,32,37], six studied dapagliflozin [21,31,33,34,36,38], and one studied canagliflozin [35].

### 3.3. Quality Assessment Results

The methodological quality of the included animal studies was assessed using a risk-of-bias checklist adapted from *The Collaborative Approach to Meta-Analysis and Review of Animal Data from Experimental Studies (CAMARADES)* [39]. Each item in the tool was rated as Yes (Y) or No (N), with Yes scored as 1 and No scored as 0. The quality score for each study was calculated as the sum of Y responses across all items, and the mean quality score was then derived from the scores of all included studies

The papers demonstrated a moderate quality of methodology, with a mean quality score of 6.83 across all 12 studies (Table 2). All were published in peer-reviewed journals and reported their adherence to relevant ethical guidelines. The studies avoided using anaesthetics that display intrinsic pharmacological activity, used appropriate animal models, and provided declarations of conflicts of interest.

Two studies did not report any temperature control as part of the husbandry conditions, a factor that may confound the assessment of animal behaviour [20,29]. All studies reported the absence of blinding during allocation, and only one study implemented blinded outcome assessment [29]. The lack of blinding in outcome assessment can introduce detection bias, which could have influenced the semi-quantitative scoring of animal parameters. Furthermore, we rated almost all studies as ‘N’ for sample size calculation. This point indicates that the experiments could have been underpowered or overpowered, which could have led to imprecise estimates of effect sizes.

### 3.4. Meta-Analysis of Morris Water Maze Results

The Morris Water Maze test is widely used to assess spatial memory function under AD-like conditions in laboratory animals. During the test, animals are required to locate a hidden platform within a set time frame. Two key metrics that are used to evaluate spatial memory in the MWM are escape latency (i.e., the time the animal takes to find the hidden platform) and the duration spent in the target quadrant during the probe trial. A shorter escape latency during the acquisition phase indicates enhanced spatial learning, whereas increased time spent in the target quadrant reflects improved memory retrieval [40].

#### 3.4.1. Escape Latency in T2dm Models

**Figure 2:**
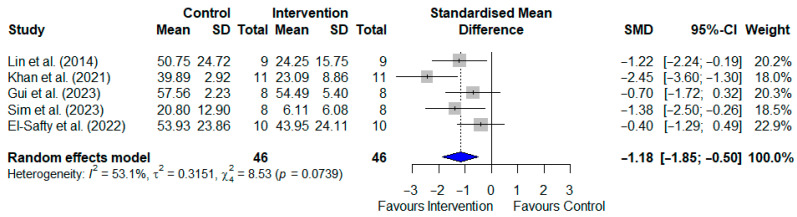
Meta-analysis of SGLT2 inhibitor effects on escape latency in the Morris Water Maze test using T2DM animal models [21,29,30,31,32]. Results are presented as standardised mean differences with 95% confidence intervals (CI). *p* < 0.05 was considered statistically significant for the overall effect.

Five studies of T2DM animal models, involving a total of 92 rodents, reported escape latency as the outcome measure [21,29,30,31,32]. Our meta-analysis showed that SGLT2i treatment significantly reduced the escape latency in the experimental groups compared to the control groups, as shown in Figure 2 [SMD: 1.12; 95% CI: (−1.56, −0.69); *p* < 0.001, I^2^ = 58.27%].

#### 3.4.2. Escape Latency in AD Models

**Figure 3:**
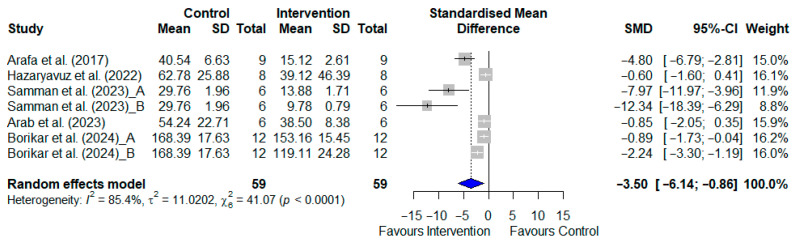
Meta-analysis of SGLT2 inhibitor effects on escape latency (Morris Water Maze) in Alzheimer’s disease animal models [33,35,36,37,38]. Data indicate the standardised mean differences with 95% confidence intervals. Samman et al. (2023) [38] tested two doses, namely 1 mg/kg (A) and 5 mg/kg (B), whereas Borikar et al. (2024) [37] tested 5 mg/kg (A) and 10 mg/kg (B). Statistical significance for the overall effects was set at *p* < 0.05.

Five studies of AD animal models, with a total of 118 rodents, reported escape latency as the outcome measure [33,35,36,37,38]. Among them, two studies tested the experimental group using two different doses [37,38]. Our meta-analysis indicated a significantly reduced escape latency in the SGLT2i-treated groups compared to the control groups, as shown in Figure 3 [SMD: −1.559; 95% CI: (−2.016, −1.103); *p* < 0.001]. However, the reported heterogeneity was high (I^2^ = 88.90%).

#### 3.4.3. Time in Target Quadrant for T2DM Models

**Figure 4:**
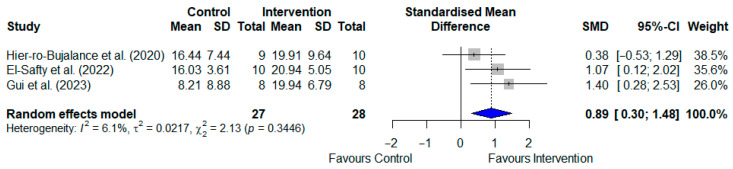
Meta-analysis of the effects of SGLT2 inhibitors on time spent in the target quadrant during the Morris Water Maze test in T2DM animal models [20,21,31]. Results are standardised mean differences with 95% confidence intervals (CI). An overall *p*-value < 0.05 was considered statistically significant.

Only three studies of T2DM animal models, involving a total of 55 rodents, examined the time spent in the target quadrant as an outcome measure [20,21,31]. The SGLT2i-treated groups showed significantly longer times in the target quadrant compared to the control groups, as shown in Figure 4 [SMD: 0.895; 95% CI: (0.345, 1.445); *p* = 0.002, I^2^ = 16.35%].

#### 3.4.4. Time in Target Quadrant for AD Models

**Figure 5:**
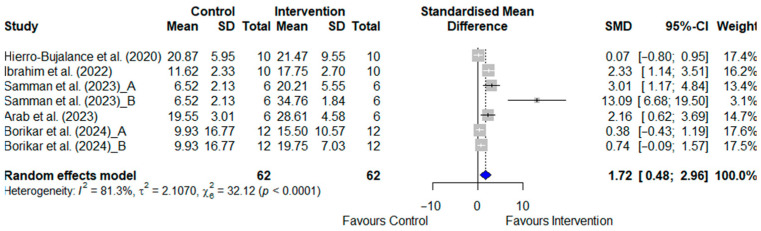
Meta-analysis of SGLT2 inhibitor effects on time spent in the target quadrant (Morris Water Maze) in Alzheimer’s disease animal models [20,34,36,37,38]. Data represent standardised mean differences (95% confidence intervals). Samman et al. (2023) [38] tested two doses, 1 mg/kg (A) and 5 mg/kg (B); Borikar et al. (2024) [37] tested 5 mg/kg (A) and 10 mg/kg (B). Statistical significance for the overall effects was set at *p* < 0.05.

Five studies of AD animal models, involving a total of 124 rodents, examined the time spent in the target quadrant as an outcome measure [20,34,36,37,38]. Two of these studies tested the experimental group with two different doses [37,38]. The SGLT2i-treated groups showed significantly longer times in the target quadrant than the control groups, as shown in Figure 5 [SMD: 1.030; 95% CI: (0.631, 1.428); *p* < 0.001]. However, the heterogeneity was high (I^2^ = 85.6%).

### 3.5. Key Molecular Outcomes

One of the principal pathological hallmarks of AD is the accumulation of Aβ in brain tissue. Neuroinflammation and oxidative stress are also recognised as major contributors to the initiation and progression of AD pathology. Although the included studies examined the effects of SGLT2 inhibitor treatment on these markers, substantial heterogeneity in their analytical methods limited these findings to descriptive summary only.

Only six studies examined changes in Aβ levels or accumulation in the brains of animal models of AD [20,36,38] and T2DM [30,31,32]. All these studies reported a reduction in Aβ accumulation in the hippocampus, except for Samman et al. (2023) [38], who observed a dose-dependent decrease in Aβ levels in whole-brain homogenates without isolating the hippocampus. Cortical Aβ levels were analysed and reported to be reduced in two studies, Hierro-Bujalance et al. (2020) [20] and Sim et al. (2023) [32]. Hierro-Bujalance et al. (2020) [20] demonstrated a significant reduction, specifically in the insoluble Aβ40 fraction. Table 3 summarises the Aβ outcomes in these AD and T2DM animal models.

Seven studies examined changes in inflammatory and oxidative stress outcomes in the brains of animal models of either AD [20,36,37,38] or T2DM [21,29,30]. All these studies reported the reduction in oxidative stress markers, specifically in the hippocampus. Two studies investigated inflammatory markers, which were also significantly decreased. Table 4 summarises these outcomes in the AD and T2DM animal models.

### 3.6. Publication Bias

A funnel plot is typically used in a meta-analysis to visually detect the presence of publication bias. Such a bias may be evident in the distribution of standard errors in individual studies. Each dot in the plot represents a study.

**Figure 6:**
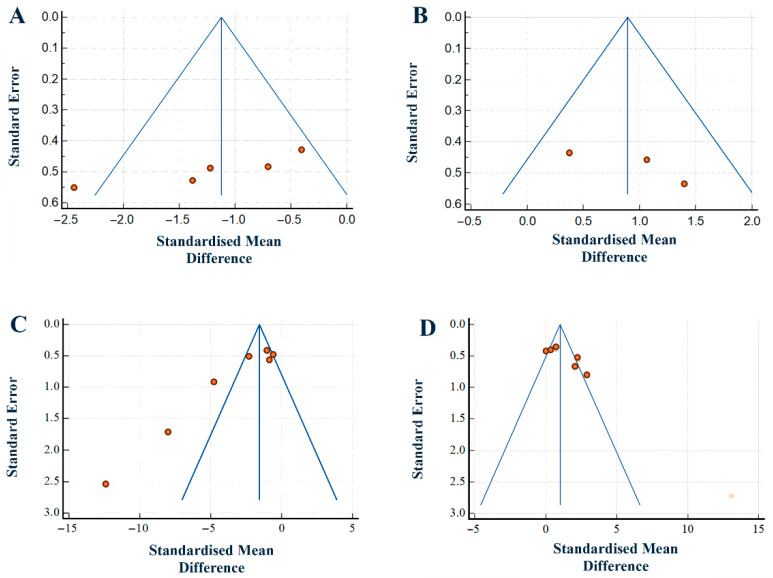
Funnel plots showing publication bias according to our meta-analyses. (**A**) Escape latency T2DM; (**B**) time spent in target quadrant T2DM; (**C**) escape latency AD; (**D**) time spent in target quadrant AD.

Our results are illustrated in Figure 6. The distributions of several studies lay outside the range of the inverted funnel plot. They included studies on escape latency in T2DM models and on escape latency and time spent in the target quadrant in AD models. Overall, the funnel plot appeared asymmetrical, which can indicate publication bias. Egger’s test was performed, and the result was *p* < 0.05, which confirms a strong likelihood of publication bias.

Across the four behavioural outcomes, trim-and-fill analysis indicated varying degrees of small-study or publication bias. For escape latency T2DM (Figure 6A) and time spent in target quadrant T2DM (Figure 6B), the procedure either imputed very few studies or none at all, implying a minimal impact of publication bias. For escape latency AD (Figure 6C) and time spent in target quadrant AD (Figure 6D), the method imputed a few missing studies in the opposite direction of the observed effects, which means the magnitude of benefit in those domains could be slightly overestimated. Importantly, in all cases, the direction of the pooled effect remained unchanged after adjustment. This result indicates that some asymmetry is present, probably due to the small number of animal studies. Our overall conclusions regarding the beneficial effects of SGLT2 inhibitors were robust. The corresponding results are presented in Supplementary Materials.

## 4. Discussion

This systematic review and meta-analysis evaluates 12 preclinical studies that investigated the effects of SGLT2i in animal models of T2DM and AD, with the aim of assessing the potential therapeutic role of SGLT2i in ameliorating memory deficits. The findings provide preliminary evidence to support the repurposing of SGLT2i for AD. Such drug repurposing could reduce development costs, shorten timelines, and increase the likelihood of therapeutic success compared with de novo development of novel agents. Our analysis indicated that SGLT2is may confer cognitive benefits by significantly enhancing spatial memory. This was evident in improved performances in the MWM test, including reduced escape latency and increased time spent in the target quadrant. Additionally, SGLT2i treatment appeared to exert anti-amyloidogenic effects in the brains of animal models, along with improvements in inflammatory markers and reduced oxidative stress.

### 4.1. Insulin Resistance Is Central in AD Development

Insulin resistance is a hallmark of T2DM and is also strongly associated with AD. This shared characteristic underscores the close interplay between metabolic and neurodegenerative disorders [41,42]. Insulin exerts neuroprotective effects in the brain, and a disruption of insulin signalling leads to neuronal damage and cognitive decline—key features in AD [43,44]. Insulin resistance adversely affects Aβ metabolism by increasing its production or impairing its clearance. It does so partly through elevated β-site amyloid precursor protein cleaving enzyme 1 (BACE1) activity, which promotes the formation of Aβ plaques [45,46,47]. Additionally, insulin resistance induces neuroinflammation, mitochondrial dysfunction, and oxidative stress, all of which contribute to the progression of AD pathology [13,48].

Interventions that target insulin resistance include SGLT2is, such as empagliflozin and dapagliflozin. These drugs have demonstrated cognitive benefits in T2DM animal models [21,32]. However, few studies have examined the effects of improved insulin sensitivity regarding cognitive function in AD animal models. Further research is warranted to determine whether enhancing insulin sensitivity could provide therapeutic benefits for patients with AD.

### 4.2. SGLT2i Improves Spatial Memory Function

SGLT2is improved the spatial memory functioning in animal models of AD and T2DM. Spatial disorientation is among the earliest clinical features of AD and is characterised by patients experiencing confusion and difficulty navigating both familiar and unfamiliar environments. This impairment often results in patients becoming disoriented, which disrupts their daily routines, compromises their independence, and poses a safety risk. One study reported that spatial disorientation affects approximately 70% of individuals with AD, with episodes ranging from single occurrences to multiple events [49].

Our meta-analysis revealed that treatment with SGLT2i significantly reduced the escape latency and increased the time spent in the target quadrant in animal models of both T2DM and AD. These preclinical findings align with previous meta-analytic evidence of improvements in cognitive function in T2DM patients with mild cognitive impairment or dementia [50]. For example, a longitudinal study reported that the use of SGLT2i for three or more years improved T2DM patients’ cognitive scores across the domains of global cognition, language, and executive function [51].

### 4.3. SGLT2i Inhibits Aβ Accumulation

The accumulation of Aβ plaques, also known as ‘senile plaques’, is a pathological hallmark of AD. These extracellular deposits of aggregated Aβ peptides disrupt neuronal functioning by several mechanisms, including synaptic dysfunction, impaired synaptic transmission and plasticity, and ultimately neuronal death [52]. Hence, Aβ accumulation correlates strongly with cognitive decline.

Based on the summary of Aβ outcomes (Table 3), all the reviewed studies reported reduced Aβ levels in either the hippocampus or the cortex of both AD and T2DM animal models after treatment with SGLT2i. The potential mechanism involves directly inhibiting the proteins involved in Aβ plaque formation. For instance, Ibrahim et al. (2022) [34] observed a 4.6-fold increase in hippocampal BACE1 levels in an ovariectomised (OVX)/D-galactose rat model of AD. The BACE1 (β-secretase) enzyme cleaves amyloid precursor protein (APP), generating a C-terminal fragment of APP (CTF99) that is further processed into Aβ [52]. Dapagliflozin administration markedly attenuated this BACE1 increase by 69% [34]. Similarly, Gui et al. (2024) [31] demonstrated that dapagliflozin reversed elevated hippocampal levels of CTF99 and BACE1 in a T2DM mouse model.

The above findings suggest that SGLT2i may affect Aβ pathology by modulating the activity of key enzymes involved in Aβ production. While the preclinical studies we reviewed provide promising evidence, human data on the effects of SGLT2i on Aβ pathology in AD are lacking. Exploring whether SGLT2i alleviates early AD pathology in amyloid-positive patients without diabetes would be a valuable direction for future research.

### 4.4. SGLT2i Targets Neuroinflammation and Oxidative Stress

Emerging preclinical studies indicate that SGLT2i targets neuroinflammation and oxidative stress, which both contribute strongly to AD progression. This evidence aligns with findings of improved spatial memory and reduced Aβ accumulation in animal models of T2DM and AD. For example, empagliflozin exhibits anti-inflammatory effects, as demonstrated by a significant reduction in hippocampal pro-inflammatory cytokines (IL-6, TNF-α, and IL-1β) in a T2DM model, as well as decreased protein expression of IL-6 and TNF-α in activated microglia. This effect probably involves inhibition of the NF-κB pathway, as demonstrated by Heimke et al. (2022) [25], who showed reduced nuclear translocation of NF-κB in LPS-stimulated microglia.

These findings suggest that SGLT2i can disrupt the pathological feedback loop of Aβ production. In that feedback loop, pro-inflammatory cytokines released from reactive microglia upregulate β-secretase expression via the NF-κB pathway. The upregulation further exacerbates Aβ generation [53].

Studies in animal models have demonstrated that SGLT2is (e.g., empagliflozin and dapagliflozin) possess antioxidant and mitochondrial protective properties. AD-related research has shown that both empagliflozin [37] and dapagliflozin [36] enhance the animal’s antioxidant defences by increasing glutathione (GSH) and catalase (CAT) levels. The drugs also upregulate key antioxidant proteins, such as nuclear factor erythroid 2-related factor 2 (Nrf2), heme oxygenase-1 (HO-1), and glutathione peroxidase (GPx).

Dapagliflozin also effectively reduces lipid peroxidation, a marker of oxidative damage [36]. Hence, SGLT2is can prevent the promotion of lipid peroxidation, which would otherwise lead to neuronal death and cognitive decline [54]. Additionally, empagliflozin [55] and dapagliflozin [31] promote mitochondrial fusion by increasing the expression of mitofusin-1 (MFN1) and mitofusin-2 (MFN2) proteins in T2DM animal models; these processes are crucial for maintaining mitochondrial integrity and function [56]. By preserving mitochondrial function, SGLT2is may thus mitigate synaptic dysfunction and cognitive decline. It is known that mitochondrial dysfunction disrupts synaptic plasticity and function [57].

### 4.5. Additional Pleiotropic Effect by SGLT2i

Interestingly, SGLT2i drugs display a dual function, acting to inhibit both acetylcholinesterase (AChE) and SGLT2. This dual action is highly relevant in AD, as the progressive degeneration of the cholinergic neuronal pathway connecting the basal forebrain to the hippocampus greatly contributes to memory impairment [58]. A molecular docking study revealed that empagliflozin exhibited a strong binding affinity to the active sites of M1 muscarinic acetylcholine receptors (mAChRs) and NMDARs, which are crucial for learning and memory functions in the hippocampus [37].

Moreover, rivastigmine (an approved AD drug) acts as an AChE inhibitor by binding to the catalytic anionic site of AChE, where ertugliflozin also binds [59,60]. Finally, canagliflozin and dapagliflozin reduce AChE activity and increase ACh levels in animal models with cognitive impairment [35,36]. This finding indicates that inhibiting AChE activity and enhancing ACh levels helps to improve cognitive function.

### 4.6. Limitations

Although a degree of neuroprotective effect of SGLT2i on AD pathology was evident from our review, it remains unclear whether the effects were directly mediated through the modulation of insulin sensitivity in the animal models. Alternatively, they might have occurred via direct inhibition of AD-related pathologies such as Tau protein development. This issue was a primary limitation of our study.

In addition, drawing definitive conclusions was challenging due to the heterogeneity among the reviewed studies, such as differences in animal models, species, age, and sex. Furthermore, the diverse methodologies employed to induce AD or T2DM, as well as variability in the SGLT2i dosing regimens and treatment durations, added to the heterogeneity.

Our Egger’s test suggested the presence of publication bias. In other words, studies reporting significant positive outcomes may have been more likely to be published than negative outcomes. This bias could potentially compromise the overall validity and generalisability of the findings presented in this review. Finally, although trim-and-fill analysis was performed, the small number of studies means that the interpretability is limited.

Overall, the findings should be interpreted with caution because of substantial heterogeneity in experimental design. Evidence of publication bias further limits the validity and interpretability of the conclusions. Thus, larger and more methodologically consistent datasets are required in future research.

## 5. Conclusions

AD is a multifactorial condition, and it is essential to develop innovative treatment strategies that address its complexity from multiple perspectives. Currently, there is a lack of safe and effective therapies that can halt or slow AD progression. Repurposing SGLT2i, which was initially developed for T2DM, shows promise in improving spatial memory deficits in both AD and T2DM animal models. The beneficial effects are potentially mediated through inhibiting Aβ plaque formation, reducing neuroinflammation and oxidative stress, and suppressing AChE activity in the brain.

Given that key pathological mechanisms are shared between T2DM and AD, SGLT2i may offer therapeutic potential for managing early AD symptoms in patients with T2DM. Furthermore, it could serve as a candidate treatment for AD itself. However, most of the current evidence is preclinical. Rigorous clinical studies, particularly randomised controlled trials in human subjects, are necessary to validate the initial findings. Such research could help to establish the efficacy of SGLT2is in mitigating the progression of AD.

## Figures and Tables

**Table 1:** Characteristics of included studies.

Study	Disease Model	Induction Method	Species	Sex	Age (Week)	Group Size		Experimental Design			Outcomes Measured		
						Ctrl	Treatment	SGLT2i	Dose	Route	Spatial Memory Function (MWM)	Aβ Markers	Inflammatory and Oxidative Stress Markers
Lin et al. (2014) [29]	T2DM	Not reported	db/db mice	M	7	9	9	EMPA	0.03%	Oral	Escape latency	Not reported	Tissue superoxide-dihydroethidium 8OHDG
Khan et al. (2021) [30]	T2DM	HFD	C57BL/6 mice	M	3–4	11	8	EMPA	4.4 mg/kg/day	Oral	Escape latency	Aβ (1–40) Aβ (1–42)	IL-6,IL-1β,TNFα SOD, catalase, TBARS
Gui et al. (2024) [31]	T2DM	STZ + HFD	C57BL/6 mice	M	6–8	10	10	DAPA	25 mg/kg/day	Oral	Escape latency Time spent in the target quadrant	Morphological changes in the hippocampus	Not reported
El-Safty et al. (2022) [21]	T2DM	STZ	Wistar rats	M	Not reported	10	10	DAPA	1 mg/kg/day	Oral	Escape latency Time spent in the target quadrant	Not reported	Not reported
Sim et al. (2023) [32]	T2DM	STZ (100 mg/kg, IP) + HFD	C57BL/6 mice	M	8	8	10	EMPA	25 mg/kg/day	Oral	Escape latency	Aβ accumulation Aβ/β actin ratio	Not reported
Hazaryavuz et al. (2022) [33]	AD	icv-STZ intracerebrovascular	Wistar rats	E	Not reported	8	8	DAPA	1 mg/kg	Oral	Escape latency	Not reported	Not reported
Samman et al. (2023) [38]	AD	AlCl_3_	Swiss rats	M	Not reported	6	6	DAPA	1 and 5 mg/kg	Oral	Escape latency	Aβ levels	MDA, SOD, CAT
Ibrahim et al. (2022) [34]	AD	Ovariectomised/D-galactose	Wistar rat	F	12–16	10	10	DAPA	1 mg/kg/day	Oral	Time spent in the target quadrant	Not reported	Not reported
Arafa et al. (2017) [35]	AD	SCO	Wistar rats	M	5–6	9	9	CANA	10 mg/kg	Oral	Escape latency	Not reported	Not reported
Borikar et al. (2024) [37]	AD	SCO	Wistar rats	M	12–16	12	12	EMPA	5 and 10 mg/kg/day	Oral	Escape latency Time spent in the target quadrant	Not reported	GSH, LPO, CAT
Arab et al. (2023) [36]	AD	CdCl_2_	Wistar rats	NR	8–10	6	6	DAPA	1 mg/kg/day	Oral	Escape latency Time spent in the target quadrant	Aβ42 expression	Not reported
Hierro-Bujalance et al. (2020) [20]	AD	Transgenic mice	APP/PS1 db/db mice	E	4	11	11	EMPA	10 mg/kg	Oral	Escape latency Time spent in the target quadrant	Soluble and insoluble Aβ40 and Aβ42	Microglia burden

Note: AD, Alzheimer’s disease; AlCl_3_, aluminium chloride; CANA, canagliflozin; CAT, catalase; EMPA, empagliflozin; DAPA, dapagliflozin; GSH, glutathione; icv-STZ, intracerebrovascular streptozocin; LPO, lipid peroxidation; MDA, malondialdehyde; SCO, scopolamine; SOD, superoxide dismutase; STZ, streptozocin; T2DM, type 2 diabetes mellitus; 8OHDG, 8-hydroxy-2′-deoxyguanosine.

**Table 2:** Our quality assessment of the reviewed studies according to the CAMARADES checklist.

No.	Author	CAMARADES Study Quality Checklist										
		(1)	(2)	(3)	(4)	(5)	(6)	(7)	(8)	(9)	(10)	Quality Score
1	Hierro-Bujalance et al. (2020) [20]	Y	N	Y	N	N	Y	Y	N	Y	Y	6
2	Lin et al. (2014) [29]	Y	N	Y	N	Y	Y	Y	N	Y	Y	7
3	Khan et al. (2021) [30]	Y	Y	N	N	N	Y	Y	Y	Y	Y	7
4	Hazaryavuz et al. (2022) [33]	Y	Y	Y	N	N	Y	Y	N	Y	Y	7
5	Samman et al. (2023) [38]	Y	Y	Y	N	N	Y	Y	N	Y	Y	7
6	Ibrahim et al. (2022) [34]	Y	Y	Y	N	N	Y	Y	N	Y	Y	7
7	Gui et al. (2024) [31]	Y	Y	Y	N	N	Y	Y	N	Y	Y	7
8	Sim et al. (2023) [32]	Y	Y	Y	N	N	Y	Y	N	Y	Y	7
9	El-Safty et al. (2022) [21]	Y	Y	Y	N	N	Y	Y	Y	Y	Y	8
10	Arafa et al. (2017) [35]	Y	Y	N	N	N	Y	Y	N	Y	Y	6
11	Borikar et al. (2024) [37]	Y	Y	N	N	N	Y	Y	N	Y	Y	6
12	Arab et al. (2023) [36]	Y	Y	Y	N	N	Y	Y	N	Y	Y	7
AVERAGE SCORE												6.83

CAMARADES checklist criteria [28]: (1) Publication in a peer-reviewed journal; (2) Statement of control of temperature; (3) Randomisation of treatment or control; (4) Allocation concealment; (5) Blinded assessment of outcome; (6) Avoidance of anaesthetics with marked intrinsic properties; (7) Use of a suitable animal model; (8) Sample size calculation; (9) Statement of compliance with regulatory requirements; (10) Statement regarding possible conflict of interest.

**Table 3:** Summary of Aβ outcomes in AD and T2DM animal models.

Study	Animal Model	Analysis Method	Effect of SGLT2i on Aβ
Hierro-Bujalance et al. (2020) [20]	AD	ELISA	Significant reduction in the level of insoluble Aβ40 in the cortex.
Arab et al. (2023) [36]	AD	ELISA	Significant reduction in the expression of Aβ42 in the hippocampus.
Samman et al. (2023) [38]	AD	ELISA	A trend of significant dose-dependent reduction in Aβ levels in the brain homogenate.
Khan et al. (2021) [30]	T2DM	ELISA	Significant reduction in hippocampus Aβ (1–40) and Aβ (1–42) levels.
		Congo red staining	Reduced Aβ accumulation in the hippocampal section.
Gui et al. (2024) [31]	T2DM	Congo red staining	Limiting the production of Aβ in the hippocampus after treatment.
Sim et al. (2023) [32]	T2DM	IHC	Lower Aβ accumulation in the cortex and hippocampus.
		WB	Lower Aβ/β actin ratio in the cortex and hippocampus.

Abbreviations: AD, Alzheimer’s disease; T2DM, type 2 diabetes mellitus; Aβ, amyloid-beta; ELISA, enzyme-linked immunosorbent assay; IHC, immunohistochemistry; SGLT2i, sodium–glucose cotransporter-2 inhibitors; WB, Western blot.

**Table 4:** Summary of inflammatory and oxidative stress outcomes in AD and T2DM animal models.

Study	Animal Model	Effect of SGLT2i on Inflammatory and Oxidative Stress
Lin et al. (2014) [29]	T2DM	Significant reduction in cerebral superoxide and DNA damage.
Khan et al. (2021) [30]	T2DM	Significantly reduced inflammatory markers (IL6, IL1B, TNFa) and oxidative stress after SGLT2i treatment. The activity of cellular antioxidant defence capacity was increased by SGLT2i treatment.
El-Safty et al. (2022) [21]	T2DM	SGLT2i treatment significantly reversed the elevation of oxidative stress in the hippocampus.
Samman et al. (2023) [38]	AD	SGLT2i treatment significantly reversed the elevation of oxidative stress in the hippocampus and improved the antioxidant markers.
Borikar et al. (2024) [37]	AD	SGLT2i treatment significantly reversed the elevation of oxidative stress in the hippocampus and improved the antioxidant markers.
Arab et al. (2023) [36]	AD	Significant reduction in the hippocampal lipid peroxide level and an elevation in the antioxidant levels.
Hierro-Bujalance et al. (2020) [20]	AD	Significant reduction in the microglia burden in the cortex and hippocampus in all conditions after SGLT2i treatment. Reductions were especially evident in the area distant from the senile plaques.

Abbreviations: AD, Alzheimer’s disease; T2DM, type 2 diabetes mellitus; SGLT2i, sodium–glucose cotransporter-2 inhibitors.

## Data Availability

The original contributions presented in this study are included in the article and Supplementary Materials. Further inquiries can be directed to the corresponding authors.

## Supplementary Materials

The following supporting information can be downloaded at https://www.mdpi.com/article/10.3390/ph19010166/s1, Supplementary Material S1: Electronic Database Strategy from inception up to December 2024; Supplementary Material S2: Trim and Fill Compiled.
