# Supplementary material for: Neuroprotective Potential of SGLT2 Inhibitors in Animal Models of Alzheimer’s Disease and Type 2 Diabetes Mellitus: A Systematic Review
Source: Pharmaceuticals (Basel). 2026 Jan 16;19(1):166. doi: 10.3390/ph19010166 (PMC12845125; doi:10.3390/ph19010166)
Supplement: Supplementary file 1 [file pharmaceuticals-19-00166-s001.zip › pharmaceuticals-4023072-supplementary.pdf]

Supplementary Material S1: Electronic Database Strategy from inception up to December 2024

Database	Terms used	Hits
Pubmed	(("sodium-glucose cotransporter-2 inhibitor"[All Fields] OR "sodium glucose cotransporter 2 inhibitor"[All Fields] OR empagliflozin[All Fields] OR dapagliflozin[All Fields] OR "SGLT2 inhibitor"[All Fields] OR SGLT2i[All Fields]) **AND** (Alzheimer[Title/Abstract] OR "Alzheimer disease"[MeSH Terms] OR "cognitive decline"[Title/Abstract] OR "memory decline"[Title/Abstract] OR "cognitive impairment"[Title/Abstract] OR "memory impairment"[Title/Abstract] OR MWMT[Title/Abstract] OR "amyloid beta"[Title/Abstract] OR "amyloid-beta"[Title/Abstract] OR "Morris water maze test"[Title/Abstract] OR "Morris water maze"[Title/Abstract] OR inflammation[Title/Abstract]) **AND** (diabetes[Title/Abstract] OR "diabetes mellitus, type 2"[MeSH Terms] OR "type 2 diabetes"[Title/Abstract] OR "insulin resistance"[Title/Abstract] OR "insulin resistance"[MeSH Terms] OR "metabolic syndrome"[Title/Abstract] OR "metabolic syndrome"[MeSH Terms]))	66
Scopus	TITLE-ABS-KEY("sodium-glucose cotransporter-2 inhibitor" OR "sodium glucose cotransporter 2 inhibitor" OR empagliflozin OR dapagliflozin OR "SGLT2 inhibitor" OR SGLT2i) **AND** TITLE-ABS-KEY(Alzheimer OR "Alzheimer disease" OR "cognitive decline" OR "memory decline" OR "cognitive impairment" OR "memory impairment" OR MWMT OR "amyloid beta" OR "amyloid-beta" OR "Morris water maze test" OR "Morris water maze" OR inflammation) **AND** TITLE-ABS-KEY(diabetes OR "diabetes mellitus" OR "type 2 diabetes" OR "type II diabetes" OR "diabetes mellitus, type 2" OR "insulin resistance" OR "metabolic syndrome")	221
Web of Science	TS=("sodium-glucose cotransporter-2 inhibitor" OR "sodium glucose cotransporter 2 inhibitor" OR empagliflozin OR dapagliflozin OR "SGLT2 inhibitor" OR SGLT2i) **AND** TS=(Alzheimer OR "Alzheimer disease" OR "cognitive decline" OR "memory decline" OR "cognitive impairment" OR "memory impairment" OR MWMT OR "amyloid beta" OR "amyloid-beta" OR "Morris water maze test" OR "Morris water maze" OR inflammation) **AND** TS=(diabetes OR "diabetes mellitus" OR "type 2 diabetes" OR "type II diabetes" OR "diabetes mellitus, type 2" OR "insulin resistance" OR "metabolic syndrome")	71

Supplementary Material S2: Trim and Fill Compiled

Escape Latency AD

Study	TE	lower_CI	upper_CI
Arafa et al. (2017)	-4.8	-6.79	-2.81
Hazaryavuz et al. (2022)	-0.6	-1.6	0.41
Samman et al. (2023)_A	-7.97	-11.97	-3.96
Samman et al. (2023)_B	-12.34	-18.39	-6.29
Arab et al. (2023)	-0.85	-2.05	0.35
Borikar et al. (2024)_A	-0.89	-1.73	-0.04
Borikar et al. (2024)_B	-2.24	-3.3	-1.19
Filled: Samman et al. (2023)_A	5.3	1.3	9.3
Filled: Samman et al. (2023)_B	9.67	3.62	15.72

Escape Latency T2DM

Study	TE	lower_CI	upper_CI
Lin et al. (2014)	-1.22	-2.24	-0.19
Khan et al. (2021)	-2.45	-3.6	-1.3
Gui et al. (2024)	-0.7	-1.72	0.32
Sim et al. (2023)	-1.38	-2.5	-0.26
El-Safty et al. (2022)	-0.4	-1.29	0.49

Time Spent T2DM

Study	TE	lower_CI	upper_CI
Hierro-Bujalance et al. (2020)	0.38	-0.53	1.29
El-Safty et al. (2022)	1.07	0.12	2.02
Gui et al. (2024)	1.4	0.28	2.53
Filled: El-Safty et al. (2022)	-0.31	-1.26	0.64
Filled: Gui et al. (2023)	-0.64	-1.76	0.49

Time Spent AD

Study	TE	lower_CI	upper_CI
Hierro-Bujalance et al. (2020)	0.07	-0.8	0.95
Ibrahim et al. (2022)	2.33	1.14	3.51
Samman et al. (2023)_A	3.01	1.17	4.84
Samman et al. (2023)_B	13.09	6.68	19.5
Arab et al. (2023)	2.16	0.62	3.69
Borikar et al. (2024)_A	0.38	-0.43	1.19
Borikar et al. (2024)_B	0.74	-0.09	1.57
Filled: Samman et al. (2023)_A	-1.41	-3.25	0.43
Filled: Samman et al. (2023)_B	-11.5	-17.91	-5.09
